# Ticking Time Bomb? Climate Change and *Ixodes scapularis*

**DOI:** 10.1289/ehp.122-A168

**Published:** 2014-06-01

**Authors:** Sharon Levy

**Affiliations:** Sharon Levy is a freelance science journalist and contributing editor to *OnEarth*, the magazine of the Natural Resources Defense Council.

Lyme disease, caused by the bacterium *Borrelia burgdorferi*, first emerged in the northeastern United States in the 1970s.^1^ Since then, the geographic range of the illness has expanded to the west, south, and north, and it has become by far the most commonly reported vector-borne disease in North America.^2^ Evidence is mounting that, on its northern front, the expanding range of Lyme disease is driven by climate change; warming temperatures allow new populations of the tick vector, *Ixodes scapularis*, to establish themselves in regions that were once too cold.^3^^,^^4^^,^^5^ Now a new study in *EHP* has quantified the relationship between warmer temperatures and the tick’s expansion into Canada.^6^

Some historical and genetic evidence suggests Lyme disease was widespread in much of the contiguous United States prior to European settlement.^7^^,^^8^ The new work shows, however, that *I. scapularis* may now be spreading into regions it never occupied before, paving the way for disease to follow. “We think that much of Canada was not suitable for ticks in the past,” says lead author Nicholas Ogden of the Public Health Agency of Canada, “even though we have the habitat and the hosts.”

**Figure:**
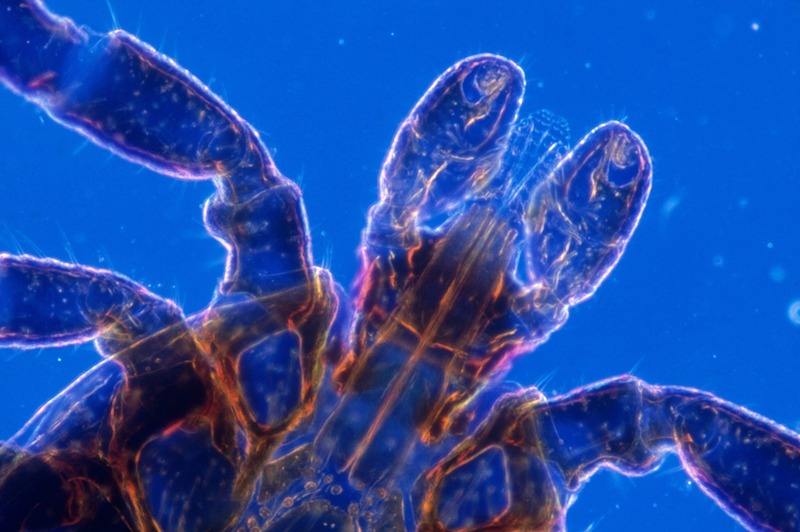
The black-legged (a.k.a. deer) tick transmits Lyme disease to humans. Removing a tick within 24 hours of being bitten can prevent transmission of the bacteria responsible for the disease. © Kent Wood/Science Source

Lyme disease is a forest phenomenon, dependent on complex relationships between its tick vector and an array of host animals that feed ticks and act as reservoirs for infection. Its emergence 30 years ago was linked to major landscape change in the northeastern United States. Regrowth of forests once cleared for farmland enabled a renaissance of the nearly eradicated white-tailed deer, a critical host for the adult life stage of the tick.^9^

Migratory birds carry infected ticks north into Canada.^3^^,^^10^ Whether the ticks survive there and establish new populations depends, to a great extent, on temperature. Newly hatched *I. scapularis* larvae need a blood meal from a host in order to progress to their next life stage as a nymph. Nymphs likewise must feed from a host to fuel their transition to adulthood, and adult females require a third blood meal in order to produce eggs.^3^

Although ticks are sheltered from freezing on the forest floor, where they spend about 90% of their 2- to 3-year life, cold temperatures mean it takes longer for an individual tick to progress through each life stage.^11^ “The longer the tick’s life cycle, the greater the proportion of ticks that will die before they reach adulthood,” explains Ogden—when temperatures are too cold, the ticks’ life cycle lengthens so much that most of them die without reproducing, and potential new populations fail to take hold.

Ogden’s new study uses biological modeling to quantify the impact of temperature on the “basic reproductive number,” denoted *R*_0_, of ticks in Canada. *R*_0_ reflects the ability of a parasite to multiply; a value greater than 1 means enough ticks will successfully reproduce to establish a viable population. The model incorporated historical and current temperature data to predict the impact of temperature increases on tick survival.

Past temperature-related shifts in *R_0_* agreed well with field data on tick populations in southern Canada and the northeastern United States, where Lyme disease was first identified. Assuming local temperature increases consistent with a global average increase of 3.4°C by 2100,^12^ the authors estimate tick *R*_0_ will increase by 2–5 times in Canada and by 1.5–2 times in the United States.

Although the current study focuses only on ticks themselves, not Lyme disease, *B. burgdorferi* typically appears in Canadian tick populations within 3–5 years of their establishment, according to Ogden. “The predicted effect of climate change on tick vectors, and therefore on disease, is quite profound,” he says. The number of reported cases of Lyme disease in Canada is already on the rise.^4^

The new study “is an important contribution to the study of climate change and vector-borne disease and makes a strong case for increased risk of Lyme disease in more northerly latitudes,” says Durland Fish, a professor of epidemiology and of forestry and environmental studies at the Yale School of Public Health. Fish coauthored a 2005 modeling study that forecast the expansion of *I. scapularis* and *B. burgdorferi* into Canada.^5^ The new work led by Ogden not only corroborates that prediction but also quantifies the estimated impacts of rising temperatures using a model that Fish describes as “an order of magnitude better” than the one he and his colleagues used a decade ago.

Fish notes limitations to the Ogden group’s model, but the tick’s life cycle is so complex that it’s difficult to gather data on every factor affecting it. “We need more study of how ticks make it—or fail to make it—in the field,” says Fish. “That’s true for all disease vectors.”
